# Distributed Acoustic Sensing (DAS) Response of Rising Taylor Bubbles in Slug Flow

**DOI:** 10.3390/s22031266

**Published:** 2022-02-07

**Authors:** Aleksei Titov, Yilin Fan, Kagan Kutun, Ge Jin

**Affiliations:** 1 Department of Geophysics, Colorado School of Mines, Golden, CO 80401, USA; gjin@mines.edu; 2 Department of Petroleum Engineering, Colorado School of Mines, Golden, CO 80401, USA; yilinfan@mines.edu (Y.F.); kutun@mines.edu (K.K.)

**Keywords:** distributed acoustic sensing (DAS), flow profiling, slug flow, Taylor bubble

## Abstract

Slug flow is one of the most common flow types encountered in surface facilities, pipelines, and wellbores. The intermittent gas phase, in the form of a Taylor bubble, followed by the liquid phase can be destructive to equipment. However, commonly used point flow sensors have significant limitations for flow analysis. Distributed acoustic sensing (DAS) can turn optical fibers into an array of distributed strain rate sensors and provide substantial insights into flow characterization. We built a 10 m vertical laboratory flow loop equipped with wrapped fiber optic cables to study the DAS response of rising Taylor bubbles. Low-passed DAS data allow for velocity tracking of Taylor bubbles of different sizes and water velocities. Moreover, we measured the velocity of the wake region following the Taylor bubble and explored the process of Taylor bubbles merging. The amplitude analysis of DAS data allows for the estimation of Taylor bubble size. We conclude that DAS is a promising tool for understanding Taylor bubble properties in a laboratory environment and monitoring destructive flow in facilities across different industries to ensure operations are safe and cost-effective.

## 1. Introduction

Flow measurements play a crucial role in understanding fluid dynamics in laboratory experiments as well as safe and economic operation of subsurface and surface facilities. Although single-phase flow metering is well-established, the development of sensors for multiphase flow evaluation is still ongoing, and currently point flow meters are in use in most cases. There is increasing demand for distributed multiphase-capable measurement solutions, both for laboratory and field environments. The need is even greater in in-well flow profiling for hydrocarbon producers. Some works suggest so-called distributed virtual flow metering based on advanced machine-learning algorithms [1,2,3]. This approach converts arrays of temperature and pressure sensors and point flowmeters to an array of distributed flowmeters. Despite significant progress in this area, the robustness of algorithms and availability of dense temperature and pressure sensors significantly limit this approach. Another solution utilizes the acoustic, strain, and temperature features of different flow types recorded by the fiber optic cable using distributed fiber optic sensing (DFOS) methods [4].

One of the most promising DFOS technologies is distributed acoustic sensing (DAS). DAS has developed rapidly in recent years. DAS utilizes telecommunication or special engineered fibers and turns them into a dense array of strain rate meters [4]. Due to the broad band of frequencies that DAS can capture, this technology has a broad spectrum of applications. For example, in the oil and gas industry, DAS applications include recording of seismic waves [5], detection of fracture propagation [6], and flow profiling [7]. In addition, DAS is sensitive to subtle temperature variations within a sub-millihertz range and can be used as distributed temperature gradient sensors (DTGS). If the sensing fiber is installed on top of a structure which can change its dimensions due to pressure change inside (e.g., pipes [8], mandrels [9]), DAS can act as a distributed pressure gradient sensor (DPGS).

From DAS data, one can measure the speed of sound in a fluid and estimate the flow rate using the Doppler effect [10,11]. Flow rate can also be calculated by mapping slugs and turbulent eddies using DAS data [7,10]. In addition, recent studies demonstrated the potential use of low frequency DAS signals to track thermal slugging in wellbores to estimate flow velocity for low-rate oil producers [12] and in a laboratory setup [13]. The implementation of these potential capabilities will highly benefit chemical, oil and gas, carbon sequestration, and geothermal industries. In this paper, we use a laboratory testing facility to advance DAS-based two-phase (liquid and gas) slug flow characterization.

Slug flow consists of intermittent liquid and gas phases [14,15]. Gas forms the Taylor bubble, which is followed by a liquid slug. Immediately after the Taylor bubble, a region with smaller dispersed bubbles forms (dispersed bubble train or wake [16]). Slug flow is one of the most common flow types encountered in wells and pipelines, which can be destructive for surface and downhole equipment. Moreover, for oil and gas industry applications, the transition to slug flow type can indicate the onset of liquid loading, leading to a shorter well life span. Hence, it is crucial to characterize such flow type, and DAS is one of the most promising solutions.

We designed and built a vertical flow loop facility to study slug flow with DAS. Our work shows that DAS can determine the velocities of both the Taylor bubble and the dispersed bubble train as well as the size of the Taylor bubble.

## 2. Data Acquisition and Processing

### 2.1. Flow Loop Testing Facility

**Figure 1:**
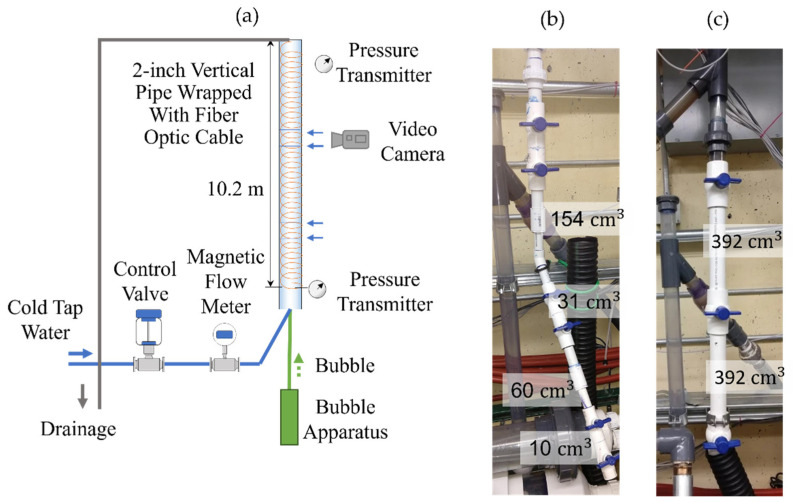
(**a**) Design of vertical flow loop facility with attached bubble apparatus. The main elements are indicated. Blue horizontal arrows point to the pipe joints. (**b**) Bubble apparatus to generate bubbles of different sizes. (**c**) Bubble apparatus to study the interaction of two bubbles of the same size.

To study slug flow in a controlled environment, we built a vertical flow loop test facility. Figure 1 shows the design of the vertical flow loop located inside a lab space with high ceilings. Telecommunication single-mode tight-buffered 900 μm fiber is wrapped around a 2-inch vertical schedule 40 PVC pipe and covers the entire length of the pipe. We found that the internal elasticity of tight-buffered fiber is sufficient for robust strain transfer in the frequency range of interest. However, to improve the strain transfer further, special adhesives used for mounting fiber Bragg gratings strain gauges can also be used. In addition, two pressure transmitters and a video camera were installed. Figure 1b shows the Taylor bubble generation apparatus, which produces air bubbles of controlled sizes. It consists of several valves connected by pipe sections. The air volume trapped between valves (at atmospheric pressure and room temperature) is released when the valves are consequently opened from top to bottom. The trapped air volume between each pair of valves used in the experiment is indicated in Figure 1b. Figure 1c presents the modification of the bubble apparatus for studying interactions between two bubbles of the same size. 

**Figure 2:**
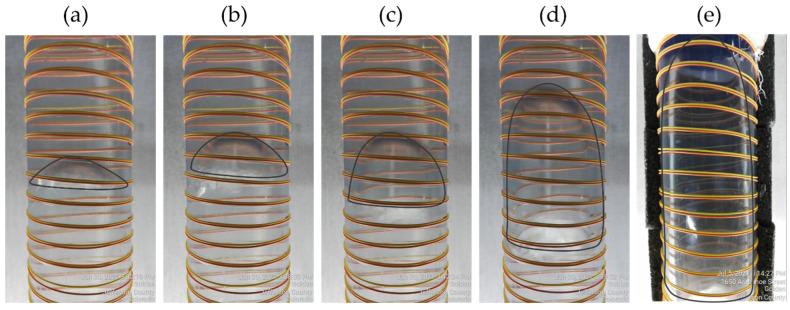
Example of generated Taylor bubbles of different sizes: (**a**) 10 ${\mathrm{cm}}^{3}$, (**b**) 31 ${\mathrm{cm}}^{3}$, (**c**) 60 ${\mathrm{cm}}^{3}$, (**d**) 154 ${\mathrm{cm}}^{3}$, and (**e**) 392 ${\mathrm{cm}}^{3}$ (the shapes are outlined in gray). The yellow tight buffered fiber was used for the DAS acquisition. The distance between neighboring wraps is 1 cm.

The generated bubbles captured by the video camera are shown in Figure 2 for all studied bubble sizes. The bubble contours are highlighted with gray lines for clarity. This figure also gives illustration of the fiber installation. The tight buffered fiber used for DAS acquisition is single-mode Corning^®^ SMF-28^®^ with a yellow jacket of $D_{f}=900\;\mu m$ in diameter. The orange fiber is a multi-mode fiber and was not used in this work. The distance ($d_{w}$) between wraps is 1 cm. The outer diameter of the PVC pipe ($D_{O}$) is 6.032 cm. Hence, to calculate the fiber-to-pipe length ratio (FPR) we use:

(1) $$FPR=\sqrt{{\left[\frac{\pi \left(D_{O}+D_{f}\right)}{d_{w}}\right]}^{2}+1}$$

where *FRP* here equals 19.26.

### 2.2. DAS Data Processing

We used the Treble interrogator unit (IU) from Terra 15 [17,18,19] to acquire the DAS data for this work. The IU natively measures and outputs the rate fiber length changes ($\dot{L}$ [m/s]) and allows for post-acquisition gauge length (GL, $L_{g}$ [m]) assignment when data are converted to strain rate ($\dot{\varepsilon }$ [(m/m)/s]) by taking spatial derivatives along the fiber axis (x):

(2) $$\;\dot{\varepsilon }\left(x\right)=\frac{\dot{L}\left(x+\frac{L_{g}}{2}\right)-\dot{L}\left(x-\frac{L_{g}}{2}\right)}{L_{g}}\;$$

**Figure 3:**
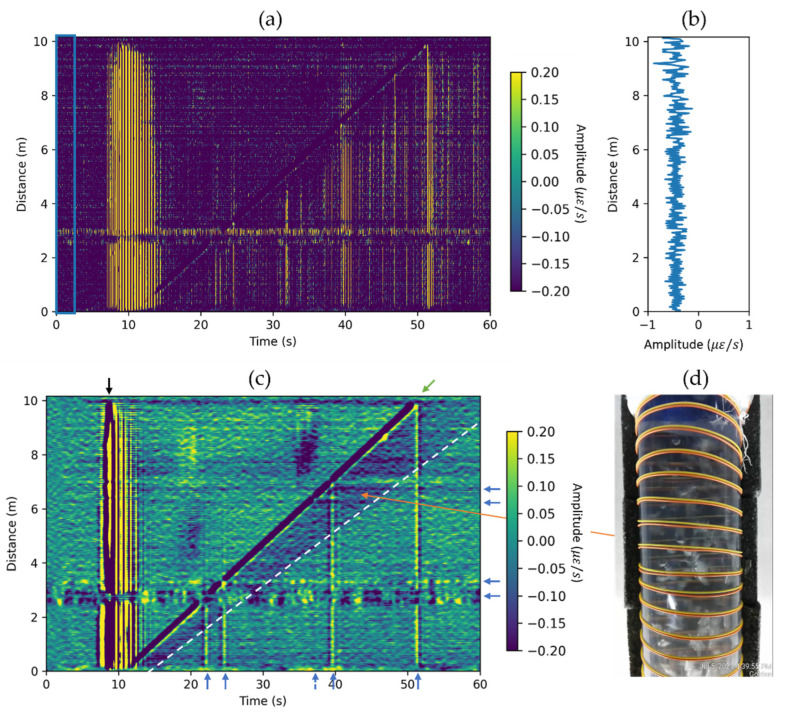
Example of DAS data processing for the Taylor bubble of $392{\;\mathrm{cm}}^{3}$ rising in the stagnant water column. (**a**) DAS data after conversion to the strain-rate domain. The baseline signal is estimated by averaging data for the first 2.5 s, as highlighted by the blue box. (**b**) The estimated baseline signal. (**c**) DAS data after baseline subtraction, application of 1 Hz low-pass filter, and ×10 downsampling. The green inclined arrow indicates the signal associated with the constantly rising velocity of Taylor bubble. The white dashed line highlights the boundary of the wake region. The black vertical arrow indicates the noise (tube waves) associated with the ball-valve opening, whereas the blue vertical arrows point to tube waves generated by the Taylor bubble passing through the pipe joints (indicated by horizontal blue arrows). (**d**) Photo of the wake region (the train of dispersed bubbles).

The optimal results are achieved when GL is equal to light pulse width (PW) sent down to fiber, which in our case was equal to 2.45 m. We followed this rule of thumb, and as our first step of data processing, we converted the raw data to strain rate, using Equation (2). The data were acquired with sampling rate $f_{s}=4\;\mathrm{kHz}$ and spatial sampling dx=0.82  m along the fiber cable. Due to wrapped fiber geometry, effective channel spacing and GL are obtained by division of fiber to pipe ratio and lead to effective channel spacing $\tilde{dx}=4.3\;\mathrm{cm}$ and effective gauge length $\tilde{L_{G}}=12.9\;\mathrm{cm}$ along the pipe length. Figure 3a shows an example of raw DAS data (waterfall plot) in terms of strain rate for a typical experiment. For all waterfall plots in this paper, the colormap limits are from −0.2 με/s to 0.2 με/s. In this experiment, the water velocity $U_{SL}=0\;m/s\;$(stagnant water column) and the bubble volume $V_{B}=392\;{\mathrm{cm}}^{3}.$ The horizontal axis is the relative time from the beginning of recording each particular experiment, and the vertical axis is the distance along the vertical pipe. The initial data have a significant amplitude shift into negative values, which is related to a slight environmental temperature decrease. The baseline signal was estimated by averaging 2.5 s (highlighted in Figure 3a with a blue box) of recorded data for each DAS channel (Figure 3b). This baseline vector was subtracted from the initial data matrix. Using frequency-wavenumber analysis, we estimated that the frequency of the signal associated with rising Taylor bubble is below 1 Hz, which is consistent with previously published computational fluid dynamics (CFD) models concerning rising Taylor bubbles [20]. Hence, in the next step, we apply a 1 Hz low-pass filter and downsample the data by a factor of ten, which led to time sampling of $\tilde{dt}=10/f_{s}=0.0025\;s$. The result of the DAS data processing is shown in Figure 3c. The signal associated with the rising Taylor bubble is clearly seen in the processed data as a diagonal dark blue line (starting around 11 s and ending around 52 s). This signal corresponds to the negative strain rate, which corresponds to a very small pipe diameter decrease as the bubble passes through. In this experiment, each DAS trace in our wrapped installation is a proxy for the pressure gradient sensor. From this figure, one can conclude that the velocity of the Taylor bubble does not change as it rises in the water column. Moreover, one can estimate velocity by fitting a linear moveout (10 m/41 s≈0.24 m/s). The automatic workflow for velocity estimation with uncertainty evaluation can be found in the next section. Observing the blue shadow following the Taylor bubble is also useful for flow analysis. The boundary between this zone and background noise is highlighted with a white dashed line. After analyzing the video record, we conclude that this blueish region is associated with a train of dispersed bubbles (wake region) following the Taylor bubble (Figure 3d). The smaller slope of the dashed white line compared with the Taylor bubble signal is associated with slower dispersed bubble train velocity (≈0.20 m/s). Other signals include tube waves, which propagate with relatively high velocity (≈1500 m/s) and result in vertical bands in the figure. The most intensive tube wave (indicated by black vertical arrow) is associated with the ball valve opening and subsequent vibration of the entire flow loop due to the mechanical impact. Other smaller tube waves are generated when the bubble hits the pipe joint or reaches the top of the water column. Horizontal blue arrows indicate the joints, whereas the corresponding tube waves are indicated by vertical blue arrows. Note that the tube waves associated with the joints mainly propagate downward, as their energy quickly dissipates in the upward direction where the Taylor bubble (gas) occupies most of the pipe cross-section.

### 2.3. Workflow for Estimating Taylor Bubble Velocity and Size

**Figure 4:**
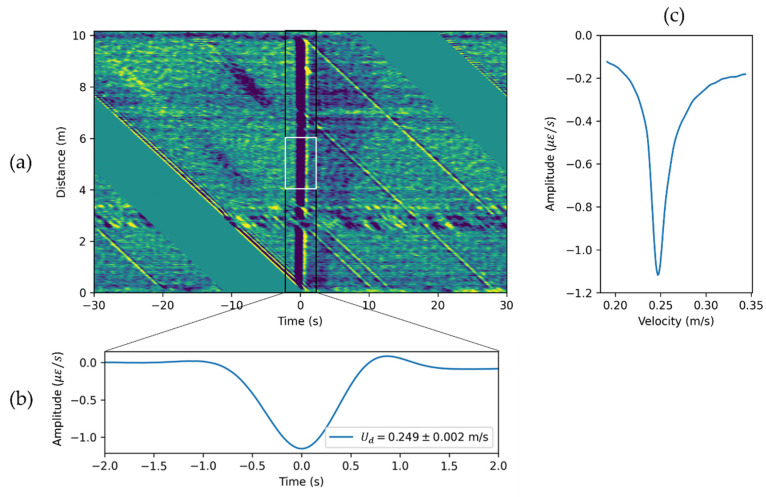
Example of Taylor bubble velocity and size estimation. (**a**) Flattened data with a velocity of 0.249 m/s. Time axis origin corresponds to the Taylor bubble signal. (**b**) DAS response averaged along distance axis zoomed in to the region of ±2 s around Taylor bubble. (**c**) Minimum averaged DAS response for flattening procedure with different velocities.

To further analyze the DAS signals associated with the Taylor bubble, we propose the following workflow. First, remove the signal related to the valve opening by assigning zeros to all DAS data from 0 s to 12 s in Figure 3b. Next, shift every subsequent channel by the number of time samples ($N_{t}$) to flatten signal associated with the Taylor bubble rise (Figure 4a). The velocity can be calculated using the following equation:

(3) $$U=\frac{\tilde{dx}\tilde{f_{s}}}{N_{t}}$$

As the Taylor bubble generates a negative strain rate response, the minimum amplitude of the averaged signal (Figure 4b) is minimized by changing velocity, as shown in Figure 4c. The minimum amplitude value in this plot corresponds to the velocity of the Taylor bubble in a stagnant water column, which is also known as drift velocity $U_{d}=0.249$ m/s.

The magnitude of the averaged signal (Figure 4b) is related to Taylor bubble size, as the larger Taylor bubble creates a larger absolute value strain rate anomaly. However, the uncertainty is high and the estimation depends on the flattening of the bubble signal. That is why for amplitude analysis, we used RMS amplitude in the ±2 s window around the Taylor bubble signal and averaged it from 4 m to 6 m in space. The window for RMS amplitude extraction is shown as a white rectangle in Figure 4a. For this example, the RMS amplitude is equal to 0.514 με/s.

## 3. Results

### 3.1. Influence of Taylor Bubble Size on DAS Response

**Figure 5:**
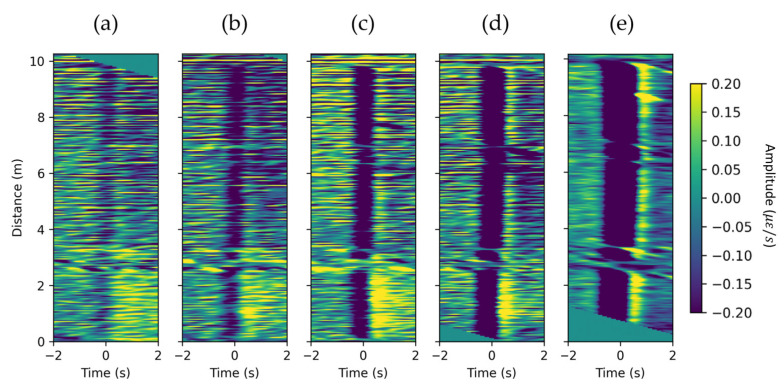
Flattened DAS response for bubbles of different volume: (**a**) 10 ${\mathrm{cm}}^{3}$, (**b**) 31 ${\mathrm{cm}}^{3}$, (**c**) 60 ${\mathrm{cm}}^{3}$, (**d**) 154 ${\mathrm{cm}}^{3}$, and (**e**) 392 ${\mathrm{cm}}^{3}$. The velocity of flattening is 0.249 m/s for every experiment.

We conducted a series of experiments to analyze how the size of generated bubbles influences DAS response. Figure 5 illustrates the flattened responses for bubbles of different sizes, shown in Figure 2. A flattening velocity of 0.249 m/s was used for all experiments. The results indicate that Taylor bubble velocity does not depend on bubble size, which is in agreement with the work of Davis and Taylor [14]. Nicklin [21] formulates that Taylor bubble velocity in a stagnant water column can be calculated using:

(4) $$U_{d}=0.35\sqrt{gD_{I}},$$

where g is the acceleration due to gravity and $D_{I}$ is the internal pipe diameter. In our case, $g=9.798\;m/s^{2}$ and $D_{I}=5.199\;\mathrm{cm}$ results in a velocity of $U_{d}=0.250\;m/s$, which is very close to the measured velocity. It is evident that the signal amplitude becomes more prominent as the volume of bubbles increase. In addition, as the time duration of the negative strain-rate disturbance broadens, it takes more time to cross a particular DAS channel for the larger bubble.

**Figure 6:**
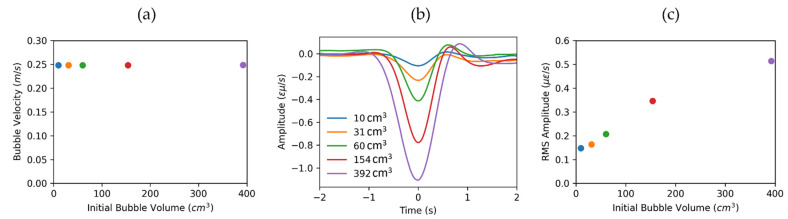
Extracted DAS data products relating to initial bubble volume. (**a**) Taylor bubble velocity is constant for all experiments $v_{B}=0.249\pm 0.002\;m/s$. (**b**) DAS response averaged along distance axis. (**c**) RMS amplitude in ±2 s window around the Taylor bubble signal from 4 m to 6 m in space.

To analyze the response more quantitatively, we utilized the proposed approaches in the previous section to extract DAS data products relating to the bubble properties. The data products relating to initial bubble volume are shown in Figure 6. Figure 6a presents the extracted velocity, which is constant for different volumes. Figure 6b shows the DAS distance-averaged response. We observed an increase in absolute amplitude perturbation and time duration of the negative region. However, as we mentioned before, a more robust way to extract associated amplitude changes is to use RMS amplitude, shown in Figure 6c. The linear fit could be a good first step for the estimation of a Taylor bubble volume. However, DAS amplitude response is rather complicated, as it involves the pipe’s response to the pressure drop associated with the bubble, mechanical coupling between the fiber and the pipe, and the effect of gauge length averaging.

### 3.2. Multiple Taylor Bubbles DAS Response in a Stagnant Water Column

**Figure 7:**
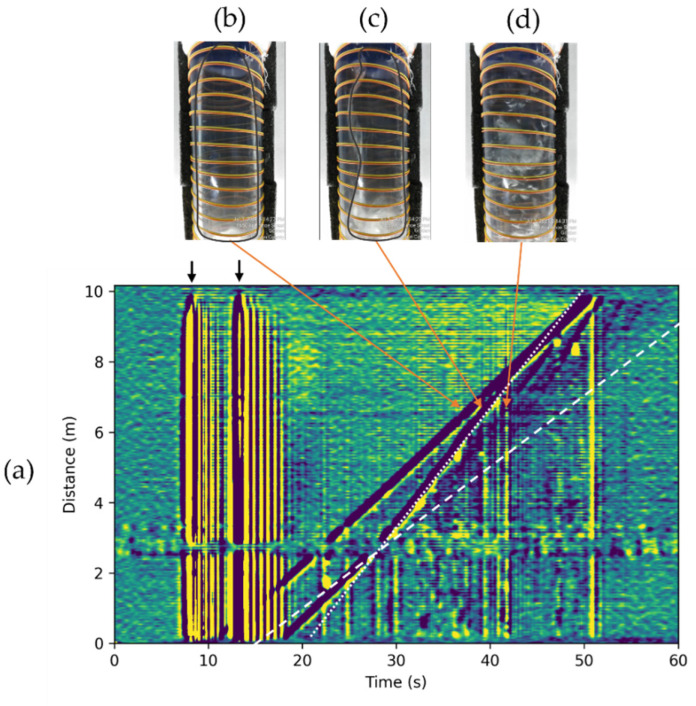
(**a**) DAS response for two 392 ${\mathrm{cm}}^{3}$ bubbles simultaneously present in a stagnant water column. Black arrows indicate the signals associated with the opening of the ball valves. The first bubble signal is similar to the signal presented in Figure 3c. The white dashed line indicates the wake region of the first Taylor bubble. The second Taylor bubble changes its velocity as soon as it arrives at the wake region of the first bubble. The velocity of the second bubble is indicated by the white dotted line. (**b**) Photo of the first Taylor bubble (shape is outlined with gray curve). (**c**) Photo of the second Taylor bubble while in the wake region of the first bubble (shape is outlined with gray curve). (**d**) Photo of a bubble in the train of the second Taylor bubble.

Due to the distributed nature of DAS measurements, this suggested workflow can be highly beneficial in studying Taylor bubble interactions, which is challenging with point sensors. The problem of Taylor bubble interactions is important in the fluid dynamics community, and a significant amount of research can be found in the literature e.g., [22]. Using the bubble apparatus (Figure 1c), we generated two bubbles of the same volume, separated by a defined length, to observe the bubble merging process in DAS data (Figure 7). In the presented experiment, bubbles were separated by 5 s (indicated by vertical black arrows), which initially equals about 1.25 m separation. From the previous experiment with a single bubble (Figure 3c), we estimated the velocity of the dispersed bubble train (wake). This is indicated by a white dashed line in Figure 7a. For the first 2 m of DAS data, both Taylor bubbles travel with the same velocity $U_{d}$; however, as soon as the second bubble intersects the wake region of the first bubble, the second Taylor bubble accelerates to a constant velocity of about 0.333 m/s (white dotted line). We validate that the observed signals are associated with the first (Figure 7b) and second (Figure 7c) bubbles using a video recording. Note that the first bubble has a bullet shape, but the turbulent wake region of the first bubble disturbs the shape of the second. However, after the bubble merging occurs, the new Taylor bubble travels at the same velocity as the first Taylor bubble. Incidentally, we noticed that more relatively large bubbles occur in the train of the second bubble (Figure 7d). The signals from them are also visible in DAS data. These bubbles are rising with the same velocity as the second Taylor bubble.

### 3.3. Influence of Water Velocity on DAS Response

**Figure 8:**
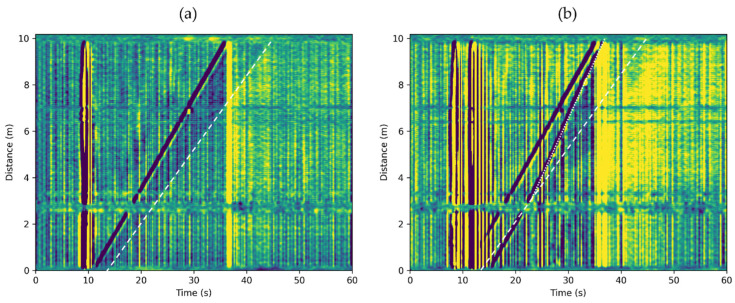
DAS response to Taylor bubbles rising in water column moving with $U_{SL}=0.096\;m/s$. (**a**) One 392 ${\mathrm{cm}}^{3}$ bubble. (**b**) Two 392 ${\mathrm{cm}}^{3}$ bubbles. White dashed lines indicate the wake region of the first Taylor bubble. The dotted white line in subplot (**b**) shows the velocity of the second Taylor bubble in the wake region of the first Taylor bubble.

In the previous sections, we analyzed the DAS response of rising Taylor bubbles in a stagnant water column. We conducted the following experiment to verify whether the suggested workflow can be applied to non-zero water superficial velocity. In the experiment, the water was flowing with $U_{SL}=0.096\;m/s$ as measured by the flow meter. *U_SL_* is the superficial liquid velocity, defined by the volumetric liquid flow rate divided by the cross-sectional area of the pipe. Figure 8a illustrates the observed DAS response to one 392 ${\mathrm{cm}}^{3}$ Taylor bubble and Figure 8b to two 392 ${\mathrm{cm}}^{3}$ bubbles present in the water column. Besides additional noise from flowing water, we still observe all the features associated with the propagation of a Taylor bubble. The Taylor bubble’s velocity in this case is $U_{T}=0.385\;m/s$.

Note that $U_{T}>U_{M}+U_{d}$, which is consistent with theoretical estimates [21]:

(5) $$U_{T}=C_{0}U_{M}+U_{d},$$

where $1.2<C_{0}<2$ is the distribution coefficient representing the impact of the velocity and concentration profile [23]. $U_{M}$ is the mixture velocity, given by

(6) $$U_{M}=U_{SL}+U_{SG},\;$$

where $U_{SG}$ is the superficial gas velocity, defined as the volumetric gas flow rate divided by the pipe cross-sectional area. In this experiment, $U_{SG}$ is negligible in Equation (6). The estimated Taylor bubble translational velocity from Equation (5) is 0.374 m/s, with a $C_{0\;}\approx 1.3$ estimated from [23]. This prediction is slightly smaller but very close to the measurement. We estimated the velocity of the second Taylor bubble after entering the wake region of the first bubble as 0.476 m/s. This velocity is higher than that in a stagnant water column by approximately 0.110 m/s, which is around 1.11 times $U_{SL}$. A coefficient larger than 1 is expected when factoring in the bubble coalescence observed at the front of the second bubble. However, more theoretical work is needed to estimate the second bubble.

## 4. Discussion and Conclusions

In this manuscript, we demonstrate that DAS is a promising tool for flow analysis, such as slug flow in the laboratory environment. After applying a low-pass filter, each DAS trace in the strain rate domain can be treated as a pressure gradient sensor, or a temperature gradient sensor, as we showed in our previous works [12,13]. Extracting velocities associated with pressure (or temperature) change is robust and can be done with high accuracy within a wide range of values. We demonstrate that it is possible to capture the velocities associated with a Taylor bubble and its wake region. Moreover, due to the distributed nature of measurements, velocity tracking can be possible along all length of the laboratory setup, allowing us to observe and analyze the process of two Taylor bubbles merging. 

DAS amplitude analysis can provide much more information regarding the measured processes. However, the DAS amplitudes should be treated carefully, as in non-invasive configuration (when the fiber is mounted on top of the piping) they are related to pressure (or temperature) variations inside a pipe as a function of DAS acquisition parameters (effective GL), pipe material, and the extent of an anomaly. If the anomaly is smaller than effective GL, the GL smoothing effect should be considered. Furthermore, inhomogeneities in fiber installation and the pipe itself can influence the strain transfer from pressure anomaly inside the pipe to the strain of the optical fiber core, which is the actual measurand. Hence, the calibration of DAS data with point sensors is necessary to achieve quantitative results. Nevertheless, we were able to extract DAS amplitudes and relate them to the Taylor bubble’s size.

The suggested fiber installation can be easily upscaled for flow monitoring in surface facilities. For example, [24] showed that installation of wrapped fiber could be used to estimate flow rate in a single-phase flow in the industrial environment based on eddy tracking and the Doppler effect. We believe that the workflow suggested in this paper can be successfully applied in field conditions, as the needed DAS sensitivity to detect the Taylor bubble is in the same order as presented in [24]. Furthermore, similar physical principles of pressure anomaly detection are possible with straight fiber installation, making the proposed methodology applicable to linear structures where the wrapped installation is sub-optimal, e.g., while flow monitoring along pipelines or in wellbores.

## Data Availability

Data and software used here are proprietary and cannot be released.
